# “They are perceived to be witches, and some are killed”: Community perceptions of elderly people living with Dementia in Kilifi, A Costal County in Kenya

**DOI:** 10.1002/alz.091291

**Published:** 2025-01-09

**Authors:** Andrew Okoth Aballa, Linda N Khakali, Willie Njoroge, Lucy Wambui Kamau, Jasmit N Shah, Karen Blackmon, Chi Udeh‐Momoh, Olivera Nesic‐Taylor, Zul Merali, Edna N Bosire

**Affiliations:** ^1^ Brain and Mind Institute, Aga Khan University, Nairobi, Nairobi Kenya; ^2^ Aga Khan University, Nairobi Kenya; ^3^ Brain and Mind Institute, Aga Khan University, Nairobi Kenya

## Abstract

**Background:**

Population growth and an increase in the number of Africans who survive to old age puts them at a higher risk of developing neurodegenerative diseases such as dementia and Alzheimer’s. Little research has been conducted on community knowledge and perceptions of dementia in rural settings in Kenya.

**Method:**

Community health volunteers, healthcare workers (HCWs), chiefs and assistant chiefs (n = 35) participated in five focus group discussions, each comprising seven‐ eight people. Key informant interviews (n = 25) were conducted with HCWs, County managers, community leaders and gatekeepers such as chiefs, traditional healers, and faith healers. Audio recordings were transcribed verbatim and thematic analysis used to extract themes.

**Result:**

Four main themes identified included: (a) understanding of dementia (b) perceived causes of dementia (c) community perceptions of dementia (d) threats exposed to elderly people suspected of having dementia. Community members did not understand the word “Dementia”. They used a local word “Kusahau sahau” meaning forgetfulness to describe dementia. People with dementia were labelled using stigmatizing words such as “*Uendawazimu‐* (*madness*”, “*craziness*”), or elderly with “*child‐like behavior*.” Dementia was perceived as a natural process of aging not requiring intervention. Witchcraftcurses, depression, trauma, substance abuse and HIV/AIDS were other factors attributed to dementia. Limited understanding of dementia led to isolation, neglect, sometimes killing of those suspected to have dementia with the assumptions they were witches.

**Conclusion:**

There is poor community understanding of dementia in rural areas of Kenya increasing stigma and discrimination for people with dementia. Retrogressive beliefs and myths predispose people with dementia to neglect, social exclusion, and violent attacks. Social and medical support programs for people with dementia and culturally acceptable tools for quickly diagnosing dementia are lacking. There is need for more research on dementia and Alzheimer’s disease, community awareness creation and policy development aimed to protect elderly populations living with Dementia.

